# An Adaptive Low-Cost INS/GNSS Tightly-Coupled Integration Architecture Based on Redundant Measurement Noise Covariance Estimation

**DOI:** 10.3390/s17092032

**Published:** 2017-09-05

**Authors:** Zheng Li, Hai Zhang, Qifan Zhou, Huan Che

**Affiliations:** 1 School of Automation Science and Electrical Engineering, Beihang University, No. 37 Xueyuan Road, Haidian District, Beijing 100191, China; lizheng@buaa.edu.cn; 2 Geomatics Engineering Department, University of Calgary, Calgary, AB T2N 1N4, Canada; 3 Space Star Technology Co., Ltd., CAST, Haidian District, Beijing 100086, China; Sarache1127@buaa.edu.cn

**Keywords:** tightly coupled navigation, measurement noise covariance estimation, adaptive Kalman filter (AKF), unscented Kalman filter (UKF), satellite selection

## Abstract

The main objective of the introduced study is to design an adaptive Inertial Navigation System/Global Navigation Satellite System (INS/GNSS) tightly-coupled integration system that can provide more reliable navigation solutions by making full use of an adaptive Kalman filter (AKF) and satellite selection algorithm. To achieve this goal, we develop a novel redundant measurement noise covariance estimation (RMNCE) theorem, which adaptively estimates measurement noise properties by analyzing the difference sequences of system measurements. The proposed RMNCE approach is then applied to design both a modified weighted satellite selection algorithm and a type of adaptive unscented Kalman filter (UKF) to improve the performance of the tightly-coupled integration system. In addition, an adaptive measurement noise covariance expanding algorithm is developed to mitigate outliers when facing heavy multipath and other harsh situations. Both semi-physical simulation and field experiments were conducted to evaluate the performance of the proposed architecture and were compared with state-of-the-art algorithms. The results validate that the RMNCE provides a significant improvement in the measurement noise covariance estimation and the proposed architecture can improve the accuracy and reliability of the INS/GNSS tightly-coupled systems. The proposed architecture can effectively limit positioning errors under conditions of poor GNSS measurement quality and outperforms all the compared schemes.

## 1. Introduction

Tightly-coupled inertial navigation system/global navigation satellite system (INS/GNSS) integration systems are an attractive positioning option in many navigation service applications [1,2]. Although considerable studies have been conducted to improve the performance or reduce the computational burden, the algorithms of the optimal adaptive filtering and satellite selection are still not theoretically and practically perfect and warrant further investigations.

A tightly-coupled system uses the GNSS pseudo-range and pseudo-range rate measurements as reference to evaluate and correct the INS error [3]. In practice, in order to cope with the unstable measurement noise covariance **R**, caused by the instabilities of the environment and the receiver [4,5], the adaptive Kalman filter (AKF) which can estimate **R** online should be utilized to guarantee the navigation accuracy [6,7,8]. The innovation and residual are the most commonly used information to estimate **R** adaptively, and the corresponding algorithms are known as innovation-based adaptive estimation (IAE) [9] and residual-based adaptive estimation (RAE) [10]. Nevertheless, the innovation and residue are coupled with the state estimation error, which can affect the accuracy of **R** and filtering or even cause divergence [11], especially in a biased state estimation situation. Besides, abnormal measurements arise in areas like urban and canyons [12]. In these areas, GNSS suffers from large errors due to the multipath, poor geometry and high noise. Dhital [13] proposed a novel adaptive filter by assuming that the measurement errors follow a heavy-tailed distribution. The user acceleration derived from GNSS Doppler measurements and the direct output of inertial measurement unit (IMU) are compared to generate a scalar to adjust **R**. Yang [14,15] introduced the robust estimation technique to INS/GNSS tightly-coupled systems to identify and reject aberrant measurements. Unfortunately, these algorithms are not theoretically quantitative and will be also affected by inaccurate state estimates such as IAE/RAE. 

Satellite selection is an important element to guarantee positioning accuracy in INS/GNSS tightly-coupled systems. Geometric dilution of precision (GDOP) [16,17,18,19,20,21], signal to noise ratio (SNR) [22,23] and carrier to noise ratio (CNR) [24,25] are standard indexes utilized to evaluate the positioning accuracy from the views of geometry constraints and signal quality. However, the GNSS measurement precision level is a crucial factor to be considered in satellite selection and has not been well studied due to a lack of effective approaches. In current GDOP- and SNR/CNR-based satellite selection algorithms, the satellites which have accurate measurement can be excluded due to low SNR/CNR or high GDOP and vice versa, the satellite suffering from large measurement error can possibly be taken into consideration to derive the navigation solution for a high SNR/CNR or low GDOP. Hence, the satellite selection algorithm faces the drawback that it may involve improper satellites or reject a suitable satellite and as such lead to negative effects on the solution. The satellite elevation angle is another factor that affects the satellite selection. The satellite elevation-dependent weight is adopted for the a priori variance for GNSS observations [26], which is based on the assumption that a lower elevation angle introduces higher measurement noise due to its increased possibility of multipath delay. However, the deficiency of lacking a real evaluation of measurement noise still exists in this approach.

To overcome the aforementioned limitations of these existing approaches, the present work proposes a novel redundant measurement noise covariance estimation (RMNCE) approach, which employs redundant information obtained from two independent measurement systems [27] to estimate their corresponding measurement noise properties. The RMNCE approach is then applied to develop a RMNCE-based tightly-coupled (RMNCE-TC) architecture, including a new RMNCE-based satellite selection algorithm and a RMNCE-based adaptive unscented Kalman filter (RMNCE-UKF). Additionally, the altitude aiding algorithm [27,28,29] is adopted to augment the external measurements for aiding the navigation solutions. The main contributions of this research are summarized below:

(1) A novel RMNCE approach is put forward and proved mathematically. The main advantage of the RMNCE approach is that the noise estimate is only based on measurements and therefore can be isolated from the state estimation error.
(2) A novel satellite selection approach is proposed by considering the measurement noise variance of different satellites, which takes both GDOP and the online estimated measurement noise into account to select an optimal satellite combination. Herein, the observation quality of the GNSS measurements can be well monitored and the differences in accuracy of different measurements can be fully considered.
(3) An AKF scheme is designed and applied to UKF [30,31]. The RMNCE-UKF ensures that the measurement noise estimate is uncorrelated to the state estimate, and correspondingly avoids the risk of filter divergence and self-oscillation. Moreover, a new **R** expansion strategy, which can be regarded as an alternative approach to Receiver Autonomous Integrity Monitoring (RAIM) and other detection algorithms, is designed to avoid the negative effects of outlying observations.

The remainder of the paper is organized as follows: Section 2 introduces the proposed RMNCE theory and provides a mathematical proof; Section 3 illustrates the proposed satellite selection algorithm; Section 4 gives the adaptive RMNCE-UKF; Section 5 presents an overview of the proposed RMNCE-TC architecture; Section 6 presents both simulation and practical test results verifying the overall system performance, and, finally, Section 7 presents the conclusions of the work.

## 2. Adaptive R Estimation

### 2.1. Related Work about **R** Estimation

The most commonly used AKF (i.e., IAE and RAE), make use of the new information in the innovation sequence or residual sequence to adaptively tune the measurement noise covariance matrix **R** [11,31]. For a nonlinear system, the two algorithms are implemented as:

(1) $$\{\begin{array}{c} {\hat{R}}_{k,IAE}=\frac{1}{N}\sum_{j=j_{0}}^{k}\varepsilon_{j}\varepsilon_{j}^{T}-P_{zz,k}^{-}\\ {\hat{R}}_{k,RAE}=\frac{1}{N}\sum_{j=j_{0}}^{k}r_{j}r_{j}^{T}+P_{zz,k}^{+} \end{array}$$

where $P_{zz,k}^{-}$ is the covariance of the predicting measurement error [31], $P_{zz,k}^{+}$ is the covariance of the filtering measurement error [32] and *N* represents the length of a sliding window. The innovation $\varepsilon_{k}$ and residue $r_{k}$ are given by:

(2) $$\{\begin{array}{l} \varepsilon_{k}=Z_{k}-h\left({\hat{X}}_{k,k-1}\right)\\ r_{k}=Z_{k}-h\left({\hat{X}}_{k,k}\right) \end{array}$$

where ${\hat{X}}_{k,k-1}$ denotes the state prediction, ${\hat{X}}_{k,k}$ denotes the state estimate and h(⋅) is the measurement function.

As shown in Equation (2), $\varepsilon_{k}$ and $r_{k}$ can be affected by biased state estimates that cannot be totally avoided in an INS/GNSS integrated navigation system. Therefore, if the system state vector is not well estimated, a negative effect will be introduced on the filter performance and as such reduce the rationality of adaptive filtering or even cause diverge. The proposed RMNCE algorithm just utilizes redundant measurements to estimate **R**, which is immune to state estimation error and then improves filtering performance.

### 2.2. RMNCE Theory

If there are two redundant measurements for the same signal with uncorrelated zero-mean white noises, the variances of the noises can be estimated just based on the measurement differences.

**Theorem.**  *Assuming that Z_1_(k) and Z_2_(k) are independent redundant measurements of a signal Z(k) from two systems, the measurements can be modeled as [27]:*

(3) $$Z_{i}\left(k\right)=Z\left(k\right)\;+\;S_{i}\left(k\right)\;+\;V_{i}\left(k\right),i=1,2$$

*where, for the measurement system i, S_i_(k) is the unknown measurement system error and V_i_(k) is zero-mean white noise at time epoch k. The first-order-self-difference (FOSD) ∆Z_i_ and the second-order-mutual-difference (SOMD) ∆Z_12_ are defined as:*

(4) $$\{\begin{array}{c} \Delta Z_{1}\left(k\right)=Z_{1}\left(k\right)-Z_{1}\left(k-1\right)\\ \Delta Z_{2}\left(k\right)\;=\;Z_{2}\left(k\right)\;-\;Z_{2}\left(k\;-\;1\right)\\ \Delta Z_{12}\left(k\right)\;=\;\Delta Z_{1}\left(k\right)\;-\;\Delta Z_{2}\left(k\right) \end{array}$$

*If S_i_(k) is stable over a short period, namely:*

(5) $$S_{i}\left(k\right)\;-\;S_{i}\left(k\;-\;1\right)\;\approx \;0$$

*the variances of V_1_(k) and V_2_(k) are given by:*

(6) $$\{\begin{array}{l} \sigma_{1}^{2}\left(k\right)=\frac{E\left[\Delta Z_{12}\left(k\right)\Delta Z_{12}^{T}\left(k\right)\right]\;+\;E\left[\Delta Z_{1}\left(k\right)\Delta Z_{1}^{T}\left(k\right)\right]\;-\;E\left[\Delta Z_{2}\left(k\right)\Delta Z_{2}^{T}\left(k\right)\right]}{4}\\ \sigma_{2}^{2}\left(k\right)\;=\;\frac{E\left[\Delta Z_{12}\left(k\right)\Delta Z_{12}^{T}\left(k\right)\right]\;-\;E\left[\Delta Z_{1}\left(k\right)\Delta Z_{1}^{T}\left(k\right)\right]\;+\;E\left[\Delta Z_{2}\left(k\right)\Delta Z_{2}^{T}\left(k\right)\right]}{4} \end{array}$$

**Proof.**  The FOSD terms of the two uncorrelated measurement systems are given by:

(7) $$\begin{array}{ccc} \Delta Z_{i}(k)=Z_{i}(k)-Z_{i}(k & -1)=\left[Z(k)\;-\;S_{i}(k)\;-\;V_{i}(k)\right]\;-\;\left[Z(k\;-\;1)\;-\;S_{i}(k\;-\;1)\;-\;V_{i}(k\;-\;1)\right] & \\ & =\left[Z(k)\;-\;Z(k\;-\;1)\right]+\;\left[S_{i}(k-1)-S_{i}(k)\right]+\left[V_{i}(k-1)-V_{i}(k)\right] & i=1,2\\ & \approx \left[Z(k)\;-\;Z(k\;-\;1)\right]+\left[V_{i}(k-1)-V_{i}(k)\right] & \end{array}$$

and the autocorrelation of $\Delta Z_{1}(k)$ can be calculated:

(8) $$\begin{array}{ll} E\left[\Delta Z_{1}(k)\Delta Z_{1}^{T}(k)\right] & =\;\left[Z(k)\;-\;Z(k\;-\;1)\right]{\left[Z(k)\;-\;Z(k\;-\;1)\right]}^{T}\;+\;E\left[V_{1}(k\;-\;1)V_{1}^{T}(k\;-\;1)\right]\;+\;E\left[V_{1}(k)V_{1}^{T}(k)\right]\\ & =\left[Z(k)-Z(k-1)\right]{\left[Z(k)-Z(k-1)\right]}^{T}+\sigma_{1}^{2}\left(k-1\right)+\sigma_{1}^{2}\left(k\right) \end{array}$$

Because the statistical characteristics are stable over a relatively short period, Equation (8) can be written as:

(9) $$E\left[\Delta Z_{1}(k)\Delta Z_{1}^{T}(k)\right]=\left[Z(k)\;-\;Z(k\;-\;1)\right]{\left[Z(k)\;-\;Z(k-1)\right]}^{T}\;+\;2\sigma_{1}^{2}\left(k\right)$$

Similarly, the autocorrelation of $\Delta Z_{2}(k)$ is given by:

(10) $$E\left[\Delta Z_{2}(k)\Delta Z_{2}^{T}(k)\right]=\left[Z(k)\;-\;Z(k\;-\;1)\right]{\left[Z(k)\;-\;Z(k\;-\;1)\right]}^{T}\;+\;2\sigma_{2}^{2}\left(k\right)$$

Considering Equations (9) and (10), we can obtain that:

(11) $$\sigma_{1}^{2}\left(k\right)\;-\;\sigma_{2}^{2}\left(k\right)=\frac{E\left[\Delta Z_{1}(k)\Delta Z_{1}^{T}(k)\right]\;-\;E\left[\Delta Z_{2}(k)\Delta Z_{2}^{T}(k)\right]}{2}$$

On the other hand, the SOMD term ∆*Z*_12_ is given by:

(12) $$\begin{array}{l} \Delta Z_{12}\left(k\right)\;=\;\Delta Z_{1}\left(k\right)\;-\;\Delta Z_{2}\left(k\right)\\ =\;\left\{\left[Z(k)\;-\;Z(k\;-\;1)\right]\;+\;\left[V_{1}(k-1)\;-\;V_{1}(k)\right]\right\}\;-\left\{\left[Z(k)-Z(k-1)\right]\;+\;\left[V_{2}(k-1)\;-\;V_{2}(k)\right]\right\}\\ =V_{1}(k\;-\;1)\;-\;V_{1}(k)\;-\;V_{2}(k\;-\;1)\;+\;V_{2}(k) \end{array}$$

and its autocorrelation is:

(13) $$\begin{array}{l} E\left[\Delta Z_{12}\left(k\right)\Delta Z_{12}^{T}\left(k\right)\right]\\ =\;E\left\{\left[V_{1}(k-1)\;-\;V_{1}(k)\;-\;V_{2}(k-1)\;+\;V_{2}(k)\right]{\left[V_{1}(k-1)\;-\;V_{1}(k)\;-\;V_{2}(k-1)\;+\;V_{2}(k)\right]}^{T}\right\}\\ =\;2\sigma_{1}^{2}\left(k\right)\;+\;2\sigma_{2}^{2}\left(k\right) \end{array}$$

Finally, by solving Equations (13) and (11), both $\sigma_{1}^{2}\left(k\right)$ and $\sigma_{2}^{2}\left(k\right)$ can be obtained as:

(14) $$\{\begin{array}{l} \sigma_{1}^{2}\left(k\right)=\frac{E\left[\Delta Z_{12}\left(k\right)\Delta Z_{12}^{T}\left(k\right)\right]\;+\;E\left[\Delta Z_{1}\left(k\right)\Delta Z_{1}^{T}\left(k\right)\right]\;-\;E\left[\Delta Z_{2}\left(k\right)\Delta Z_{2}^{T}\left(k\right)\right]}{4}\\ \sigma_{2}^{2}\left(k\right)=\frac{E\left[\Delta Z_{12}\left(k\right)\Delta Z_{12}^{T}\left(k\right)\right]\;-\;E\left[\Delta Z_{1}\left(k\right)\Delta Z_{1}^{T}\left(k\right)\right]\;+\;E\left[\Delta Z_{2}\left(k\right)\Delta Z_{2}^{T}\left(k\right)\right]}{4} \end{array}$$

☐

From the above derivation, the advantages of RMNCE algorithm can be summarized as below:

(1) The estimate of variance is only based on measurements and therefore can be isolated from the state estimation error.
(2) The estimate of variance is immune to measurement system errors and can be determined without any knowledge of the real measurement by applying FOSD and SOMD.
(3) The noise variances of redundant measurements can be estimated simultaneously.

In practice, because the statistical characteristics are stable over a relatively short period, a sliding window [27] can be employed to derive the autocorrelations in Equation (14). The sliding window width can be empirically set to 30~60.

## 3. RMNCE-Based Satellite Selection

Optimal satellite selection is an important way to realize acceptable accuracy with minimum computation [33]. The tightly coupled algorithm approach has been proven to be an effective method of positioning based on limited satellites, especially in harsh situations [34,35]. For an embedded tightly coupled system, proper satellite selection, based on indexes to evaluate measurement accuracy directly, plays an important role to guarantee filtering precision with necessary calculations.

### 3.1. Deficiency of GDOP Based Methods

After the compensation of satellite clock offset, ionospheric delay and tropospheric delay, the pseudo-range observation model [36] is reduced to:

(15) $$\rho_{GNSS}=\sqrt{{\left(x_{s}\;-\;x\right)}^{2}+{\left(y_{s}-\;y\right)}^{2}+{\left(z_{s}-z\right)}^{2}}\;+\;\delta t_{u}\;+\;\Delta \rho$$

where ${\left[x_{s},y_{s},z_{s}\right]}^{T}$ is the position of the satellite, ${\left[x,y,z\right]}^{T}$ is the position of receiver, $\delta t_{u}$ is the receiver clock offset delay, and Δρ denotes the measurement noise. Meanwhile, replacing ${\left[x,y,z\right]}^{T}$ with the INS result ${\left[x_{INS},y_{INS},z_{INS}\right]}^{T}$, a prediction of the GNSS observation, which can be taken as a redundant measurement, can be derived and denoted as $\rho_{INS}$. Because the INS and the GNSS are two independent systems, the measurement noise of $\rho_{GNSS}$ and $\rho_{INS}$ are uncorrelated. By using the RMNCE approach the variance of Δρ can be estimated as ${\hat{\sigma }}_{\rho }^{2}$. Similarly, the measurement noise variance ${\hat{\sigma }}_{\dot{\rho }}^{2}$ of the pseudo-range rate can be estimated simultaneously.

According to the measurement of pseudo-range Equation (15), the GNSS positioning accuracy can be analyzed as below:

(16) $$\Delta x={\left(H^{T}H\right)}^{-1}H^{T}\Delta \rho ,\;H=\left[\begin{array}{cccc} \frac{\partial \rho_{1}}{\partial x} & \frac{\partial \rho_{1}}{\partial y} & \frac{\partial \rho_{1}}{\partial z} & 1\\ \frac{\partial \rho_{2}}{\partial x} & \frac{\partial \rho_{2}}{\partial y} & \frac{\partial \rho_{2}}{\partial z} & 1\\ \vdots & \vdots & \vdots & 1\\ \frac{\partial \rho_{n}}{\partial x} & \frac{\partial \rho_{n}}{\partial y} & \frac{\partial \rho_{n}}{\partial z} & 1 \end{array}\right]$$

where Δx is the error vector of the receiver and H is the Jacobin matrix with respect to the expanded vector ${\left[x,y,z,\delta t_{u}\right]}^{T}$. $\mathrm{GDOP}=\sqrt{\mathrm{trace}\left\{{\left(H^{T}H\right)}^{-1}\right\}}$ is the traditional index to select the optimal satellite combination. For embedded systems, because the directions of the satellites in the sky change slowly, H can be periodically updated to decrease the computational burden.

In order to consider other factors besides geometry, weighted GDOP defined as below based on prior knowledge has been implemented and proved more effective than standard GDOP:

(17) $${\mathrm{GDOP}}_{W}=\sqrt{\mathrm{trace}\left\{{\left(H^{T}WH\right)}^{-1}\right\}}$$

Although weighted GDOP and SNR/CNR have been utilized to select optimal satellites in previous research, the uncertainty of weight and the inconsistency between the SNR/CNR and the measurement accuracy will degrade the effectiveness. The exclusion method using elevation and SNR/CNR has the risk of selecting the satellites with bad quality or missing the satellites with good quality. Hence, if the accuracy of pseudo-range can be evaluated precisely in real time, the selection reasonability and the subsequent positioning accuracy can be improved.

### 3.2. RMNCE Based Method

As described in Section 3.1, the RMNCE is employed to estimate the measurement noise variances of the pseudo-range and pseudo-range rate. Assuming that the number of satellites at epoch *k* is $S_{N}$, the estimate of $R_{k}$ can be expressed in the following form:

(18) $${\hat{R}}_{k}^{RMNCE}=diag\left\{{\hat{\sigma }}_{\rho ,1}^{2},{\hat{\sigma }}_{\rho ,2}^{2},\cdots ,{\hat{\sigma }}_{\rho ,S_{N}}^{2},{\hat{\sigma }}_{\dot{\rho },1}^{2},{\hat{\sigma }}_{\dot{\rho },2}^{2},\cdots ,{\hat{\sigma }}_{\dot{\rho },S_{N}}^{2}\right\}$$

where ${\hat{\sigma }}_{\rho ,l}^{2}$ and ${\hat{\sigma }}_{\dot{\rho },l}^{2}$ respectively denote the estimated variance of the pseudo-range and pseudo-range rate measurement noise of the *l*-th satellite.

Since pseudo range can be measured from INS and GNSS simultaneously, the GNSS measurement noises are able to be evaluated by RMNCE without the interference of state estimation error coming from IAE or RAE. At each epoch ${\hat{\sigma }}_{\rho }^{2}\left(k\right)$ is updated, then the selection operations can be expressed as follows:

(19) $$\Xi_{1}=\left\{S_{l}|\;\left|\rho_{GNSS}^{l}\left(k\right)\;-\;\rho_{INS}^{l}\left(k\right)\right|\;\leq \;\Delta \rho_{thrd}\;\mathrm{and}\;{\hat{\sigma }}_{S_{l}}^{2}\left(k\right)\;\leq \;{\hat{\sigma }}_{thd}^{2},S_{l}\in S(k)\right\}$$

(20) $$W=\left\{w_{l}|\frac{1}{{\hat{\sigma }}_{S_{l}}^{2}},S_{l}\in \Xi_{1}\right\}$$

(21) $$\Xi_{2}=\left\{S_{l}^{\prime }|\;{\mathrm{GDOP}}_{W}=\min,S_{l}^{\prime }\in \Xi_{1}\right\}$$

where S(k) is the set of observable satellites at epoch *k*, $\Delta \rho_{thrd}$ is the threshold of pseudo range difference between INS and GPS, ${\hat{\sigma }}_{thd}^{2}$ is the threshold of variance, $\Xi_{1}$ is the candidate set discarding the abnormal observations, and $\Xi_{2}$ is the selection set. 

In Equation (19), the pseudo range difference between INS and GNSS is taken as an index to reject abnormal pseudo-range errors mainly caused by the multipath effect [37]. However, this index is biased because it is affected by the INS positioning error, which may cause the wrong detection. To cope with this problem, as shown in Equation (19), satellite measurement noise variances estimated by RMNCE are considered with the pseudo range difference together to detect the abnormal measurements. Moreover, as illustrated in Equations (20) and (21), the weighting matrix **W** which is calculated by the estimated noise variances $\left\{{\hat{\sigma }}_{S_{l}}^{2}\right\}$ is used to derive the weighted GDOP; thus, both satellite geometry and measurement noise characteristic have been taken into consideration in the satellite selection procedure.

**Figure 1:**
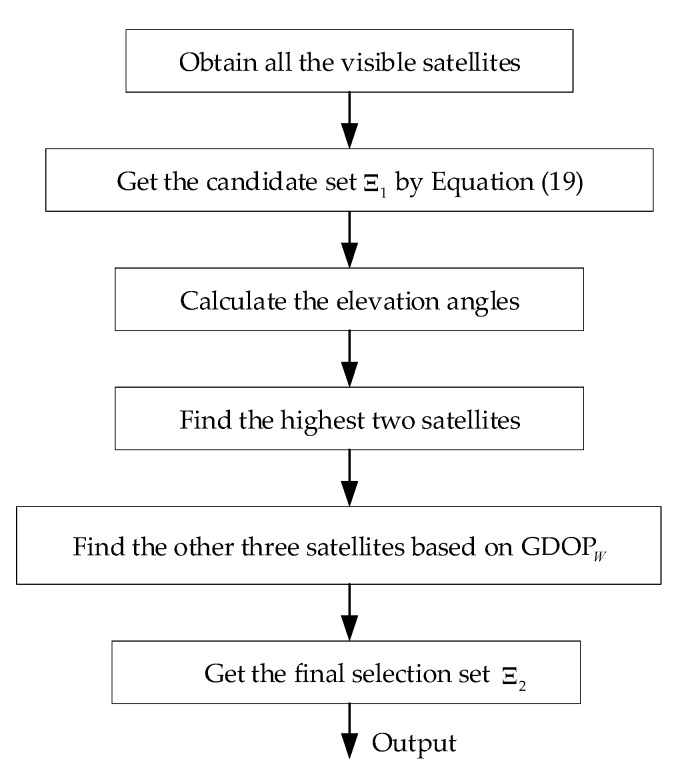
The flow chart of the proposed satellite selection.

Besides, five satellites, when available, are selected to improve the positing accuracy in this research. In order to speed up processing, all the observable satellites are sorted in descending order according to elevation angle, and the first two as mentioned in [33] are selected. Then the remained satellites can be determined according to ${\mathrm{GDOP}}_{W}$. The main procedure of the proposed satellite selection algorithm is shown in Figure 1.

**Remark** **1.** *In Equation (15), the receiver clock offset delay is unimportant when only implementing the RMNCE method, because this term cancels in the SOMD. However, the satellite exclusion step described by Equation (19) requires that* $\rho_{GNSS}$ *and* $\rho_{INS}$ *should be comparable. Hence, the compensated* $\rho_{GNSS}$ *not the direct measurement is used in Equation (19).*

## 4. RMNCE-Based Adaptive UKF

In this work, nonlinear system state model and nonlinear measurement model are employed to achieve better performance, and the RMNCE approach is applied to design an adaptive UKF. Furthermore, a **R** expanding strategy is proposed to inhibit the negative effects of the sudden enlargement of the measurement noise and abnormal observations, that always happens with multipath effect and cannot be tackled by the statistic RMNCE method.

### 4.1. INS/GNSS System Description

The state vector in the proposed tightly-coupled system is given by:

(22) $$X={\left[\phi_{E},\phi_{N},\phi_{U},\delta V_{E},\delta V_{N},\delta V_{U},\delta L,\delta \lambda ,\delta h,\varepsilon_{x},\varepsilon_{y},\varepsilon_{z},\nabla_{x},\nabla_{y},\nabla_{z},\delta t_{u},\delta {\dot{t}}_{u}\right]}^{T}$$

where ${\left[\phi_{E},\phi_{N},\phi_{U}\right]}^{T}$ is the misalignment vector between the true and estimated local navigation frame in East-North-Up (ENU) coordinate, ${\left[\delta V_{E},\delta V_{N},\delta V_{U}\right]}^{T}$ is the velocity error, ${\left[\delta L,\delta \lambda ,\delta h\right]}^{T}$ is the position error, ${\left[\varepsilon_{x},\varepsilon_{y},\varepsilon_{z}\right]}^{T}$ is the gyroscope bias, ${\left[\nabla_{x},\nabla_{y},\nabla_{z}\right]}^{T}$ is the accelerometer bias, $\delta t_{u}$ and $\delta {\dot{t}}_{u}$ are respectively the GNSS receiver clock offset and clock drift. The nonlinear differential equations of INS states are well known and can be found in [38,39,40]. The biases of IMU sensors are considered constant, and $\delta {\dot{t}}_{u}$ is modeled as a first-order Gauss-Markov process.

The measurement equation of pseudo-range is shown in Equation (15). The pseudo-range rate observation model is formulated as:

(23) $${\dot{\rho }}_{GNSS}=e_{x}\left(v_{s,x}-v_{x}\right)+e_{y}\left(v_{s,y}-v_{y}\right)+e_{z}\left(v_{s,z}-v_{z}\right)+\delta {\dot{t}}_{u}\;+\;\Delta \dot{\rho }$$

where ${\left[v_{s,x},v_{s,y},v_{s,z}\right]}^{T}$ is the satellite velocity, ${\left[v_{x},v_{y},v_{z}\right]}^{T}$ is the receiver’s velocity, ${\left[e_{x},e_{y},e_{z}\right]}^{T}$ is the unit vector between the receiver and the satellite, $\delta {\dot{t}}_{u}$ denotes the receiver clock drift and $\Delta \dot{\rho }$ is the measurement noise of pseudo-range rate.

Besides, altitude information is employed to augment the observation system. The ellipsoid equation is employed to serve as an auxiliary measurement equation [27]:

(24) $$\frac{x^{2}}{{\left(R_{e}+h\right)}^{2}}\;+\;\frac{y^{2}}{{\left(R_{e}+h\right)}^{2}}\;+\;\frac{z^{2}}{{\left(R_{p}+h\right)}^{2}}=1$$

where *h* is the altitude information, $R_{e}$ and $R_{p}$ are the lengths of the Earth’s semi-major and semi-minor axes respectively.

### 4.2. Expanded **R** Design

${\hat{R}}_{k}^{RMNCE}$ is a statistic within a sliding window and therefore cannot keep up with the change of **R** in real time, which will lead to improper estimate especially in case of multipath, disturbance and other sudden measurement noise changing circumstances. To cope with this indeterminate **R**, a performance based **R** expanding algorithm is designed as below.

Assuming that the expanded ${\bar{R}}_{k}$ can be defined by:

(25) $${\bar{R}}_{k}=\beta {\hat{R}}_{k}^{RMNCE}$$

where β is the expanding scale and can be expressed as $diag\left\{\beta_{1}\cdots \beta_{2S_{N}}\right\}$. Then the estimated state vector ${\hat{X}}_{k}$ in the UKF can be expressed as:

(26) $$\{\begin{array}{l} {\hat{X}}_{k}={\hat{X}}_{k}^{-}+K_{k}\varepsilon_{k}\\ K_{k}=P_{xz,k}{\left(P_{{\hat{z}}^{-},k}\;+\;\beta {\hat{R}}_{k}^{RMNCE}\right)}^{-1} \end{array}$$

where ${\hat{X}}_{k}^{-}$ is the state prediction, $P_{xz,k}$ is the covariance between ${\hat{X}}_{k}^{-}$ and the measurement prediction, and $P_{{\hat{z}}^{-},k}$ is the covariance of the measurement prediction error. To control the influence of the undetermined measurement noise, we assume that $K_{k}\varepsilon_{k}$ should lie within a reasonable range:

(27) $$P_{xz,k}{\left(P_{{\hat{z}}^{-},k}+\beta {\hat{R}}_{k}^{RMNCE}\right)}^{-1}\varepsilon_{k}\leq C$$

where C denotes the maximum state transition error and processing noise in one step. For an INS/GNSS integrated navigation system, **C** is mainly decided by the performance of IMU.

In order to avoid the computational complexity, sequential processing [41] is employed to estimate the boundary value of β. In the *l*-th step of the sequential UKF at the *k*-th epoch, the corresponding expanding scale $\beta_{l}$ can be calculated as:

(28) $$\{\begin{array}{c} \beta_{l}=\left[\frac{P_{xz,k}^{\left(l\right)}{}^{T}P_{xz,k}^{\left(l\right)}}{P_{xz,k}^{\left(l\right)}{}^{T}C}\varepsilon_{k}^{\left(l\right)}\;-\;P_{{\hat{z}}^{-},k}^{\left(l\right)}\right]\frac{1}{{\hat{R}}_{k}^{RMNCE,(l)}}\\ P_{xz,k}^{\left(l\right)}={\sum_{i=0}^{2n}w_{\left(i\right)}^{\left(c\right)}\left(\chi_{k,\left(i\right)}^{\left(l\right)}-{\hat{X}}_{k}^{-(l)}\right)\left\{h^{\left(l\right)}\left[\chi_{k,\left(i\right)}^{\left(l\right)}\right]\;-\;\sum_{i=0}^{2n}\omega_{\left(i\right)}^{\left(m\right)}h^{\left(l\right)}\left[\chi_{k,\left(i\right)}^{\left(l\right)}\right]\right\}}^{T}\\ P_{{\hat{z}}^{-},k}^{\left(l\right)}=\sum_{i=0}^{2n}\omega_{\left(i\right)}^{\left(c\right)}\left\{h^{\left(l\right)}\left[\chi_{k,\left(i\right)}^{\left(l\right)}\right]\;-\;\sum_{i=0}^{2n}\omega_{\left(i\right)}^{\left(m\right)}h^{\left(l\right)}\left[\chi_{k,\left(i\right)}^{\left(l\right)}\right]\right\}{\left\{h^{\left(l\right)}\left[\chi_{k,\left(i\right)}^{\left(l\right)}\right]\;-\;\sum_{i=0}^{2n}\omega_{i}^{\left(m\right)}h^{\left(l\right)}\left[\chi_{k,\left(i\right)}^{\left(l\right)}\right]\right\}}^{T} \end{array}$$

where $\varepsilon_{k}^{\left(l\right)}$ is the innovation of the *l*-th observation, $\left\{\omega_{\left(i\right)}^{\left(m\right)}\right\}$ and $\left\{\omega_{\left(i\right)}^{\left(c\right)}\right\}$ are UT weights [30], $\left\{\chi_{k,\left(i\right)}^{\left(l\right)}\right\}$ denotes the sigma points, ${\hat{X}}_{k}^{-(l)}$ denotes the prediction in the *l*-th step, $h^{\left(l\right)}\left[\cdot \right]$ is the *l*-th observation equation, $P_{xz,k}^{\left(l\right)}$ is a vector and $P_{{\hat{z}}^{-},k}^{\left(l\right)}$ is a scalar quantity.

According to the performance vector **C** and innovation, the expanding scale of every measurement can be determined but still not optimal due to the insufficient samples. For this improved tightly coupled application, the maximum expanding scale is selected as the unique scale for those abnormal measurements to suppressing undetected measurement noise effectively:

(29) $$\{\begin{array}{lll} \beta_{l}=\beta_{l+S_{N}}= & \max\{\beta_{i}|i\;=\;1,2,\cdots ,S_{2S_{N}} & \},\left|\rho_{GNSS}^{l}\left(k\right)\;-\;\rho_{INS}^{l}\left(k\right)\right|\;\geq \;\Delta \rho_{thrd}\\ \beta_{l}=\beta_{l+S_{N}}=1 & & ,\left|\rho_{GNSS}^{l}\left(k\right)\;-\;\rho_{INS}^{l}\left(k\right)\right|\;\leq \Delta \rho_{thrd} \end{array}$$

where $\beta_{l}$ and $\beta_{l+S_{N}}$ are the expanding scales corresponding to the pseudo-range and pseudo-range rate of the *l*-th satellite. The same $\Delta \rho_{thrd}$ in Equation (19) is utilized to judge whether expanding or not when abnormal measurements must be selected in challenging situations.

Regarding the particularity of inertia based integrated navigation system, this strategy is proved effective to cope with multipath and other GNSS challenging situations. In practice, the transition, input and output matrixes are usually stable or tiny different in a given period, the expanding scale can be determined based on the experiment data.

**Remark** **2.** *The threshold value* C *should be time varying due to the INS cumulative error, and increases with δt, which is the difference between the current epoch and the epoch at which the previous feedback correction was implemented. In this paper,* C *is set to be linearly increasing with respect to δt.*

### 4.3. Application in UKF

**Figure 2:**
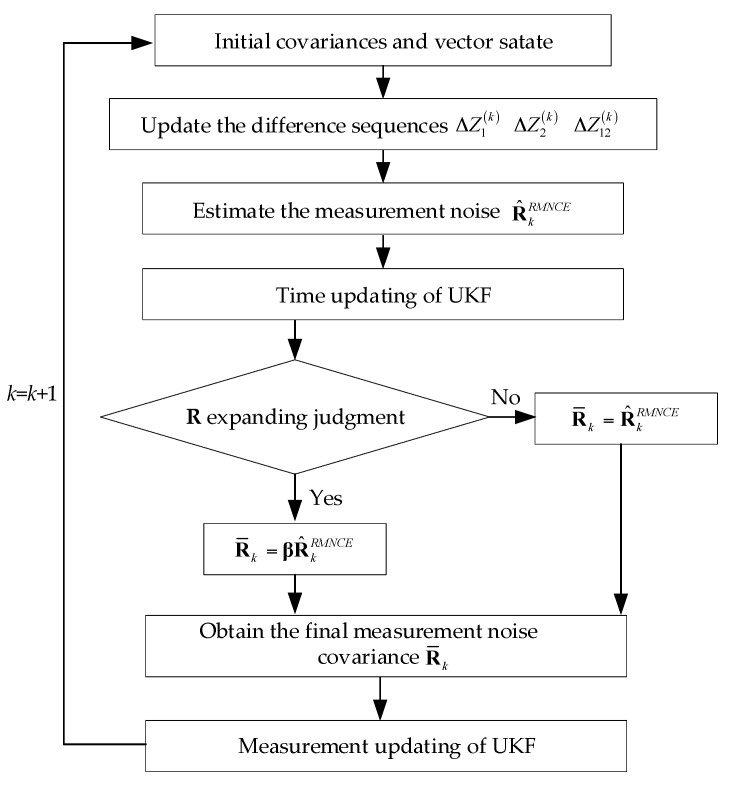
The flow chart of the proposed adaptive RMNCE-UKF.

The aforementioned adaptive strategy is employed to the UKF to solve the nonlinear estimation problem. Omitting the well-known details of the UKF, the main procedure of calculating the Kalman gain in the proposed RMNCE-UKF is shown from Equation (30) to Equation (33):

(30) $$K_{k}=P_{xz,k}P_{zz,k}^{-1}$$

(31) $$P_{xz,k}=\sum_{j=1}^{2N+1}w_{j}^{\left(c\right)}\left(\chi_{k,j}^{*}\;-\;{\hat{X}}_{k}^{-}\right){\left(\varsigma_{k,j}^{*\;-}\;-\;{\hat{Z}}_{k}^{-}\right)}^{T}$$

(32) $$P_{zz,k}=P_{{\hat{z}}^{-},k}\;+\;\beta {\hat{R}}_{k}^{RMNCE}$$

(33) $$P_{{\hat{z}}^{-},k+1}=\sum_{j=1}^{2N+1}w_{j}^{\left(c\right)}\left(\varsigma_{k,j}^{*\;-}\;-\;{\hat{Z}}_{k}^{-}\right){\left(\varsigma_{k,j}^{*}\;-\;{\hat{Z}}_{k}^{-}\right)}^{T}$$

where $\left\{\chi_{k-1,j}^{*}\right\}$ and $\left\{\varsigma_{k+1,j}^{*\;-}\right\}$ are the unscented transformations of the sigma points regarding to the state equation and measurement equation respectively, ${\hat{X}}_{k}^{-}$ is the state prediction and ${\hat{Z}}_{k}^{-}$ is the measurement prediction. The detailed time updating and measurement updating can be found in [31]. Figure 2 presents the flow chart of the RMNCE-UKF.

## 5. RMNCE-TC Architecture Overview

**Figure 3:**
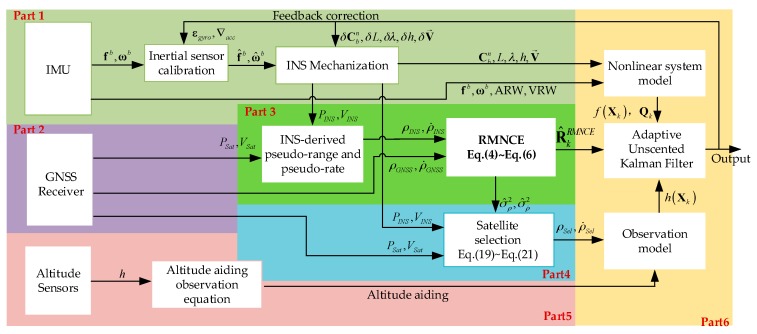
The proposed tightly-coupled architecture based on the RMNCE approach.

Figure 3 depicts the proposed architecture and illustrates how the proposed RMNCE approach forms the core component linking the INS, the adaptive UKF, and the satellite selection processes.

As shown in Figure 3, the modified tightly-coupled system consists of 6 parts. Part 1 and Part 2 are traditional INS and GNSS procedures. In Part 3, the RMNCE is carried out based on the pseudo ranges and pseudo range rates inputted by INS and GNSS according to Equations (4)~(6), which is the key adaptive part of this architecture. Satellite selection is accomplished in Part 4 based on Equations (19)–(21) and the result is also transferred to Part 6 to expand the **R** when necessary. Part 5 provides additional height observation to enhance state observabilities. The adaptive UKF is carried out in Part 6 based on RMNCE and expanded **R**.

## 6. Experiments and Discussion

Both semi-physical simulation and field experiments were conducted to evaluate the performance of the RMNCE-TC architecture.

### 6.1. Description of the Algorithms Employed for Comparison

Many researches [42,43] show that the UKF has a higher accuracy than the EKF, so we directly employ existing adaptive UKF schemes to conduct the comparison experiments. All the compared schemes with the same altitude aiding strategy [27] are briefly described below:

(1) Standard tightly-coupled integration (STC)

A standard UKF integration filter is used to fuse INS results and GNSS measurements, and **R** is set to a fixed value. Four satellites are selected by picking their GDOP ranked least.

(2) Adaptive tightly-coupled integration (ATC)

Residual-based adaptive estimation (RAE) is implemented to improve the performance of the UKF. For the RAE-UKF scheme [31], **R** is adaptively updated as follows:

(34) $$\{\begin{array}{l} {\hat{R}}_{k}={\hat{C}}_{vk}\;+\;P_{zz,k}^{+}\\ {\hat{C}}_{vk}=\frac{1}{N}\sum_{j=j_{0}}^{k}r_{j}r_{j}^{T}\\ r_{j}=Z_{j}\;-\;\sum_{i=0}^{2n}\omega_{\left(i\right)}^{\left(m\right)}h\left[\chi_{j,(i)}^{}\right]\\ P_{zz,k}^{+}=\sum_{i=0}^{2n}\omega_{\left(i\right)}^{\left(c\right)}\left\{h\left[\chi_{k,(i)}^{}\right]\;-\;\sum_{i=0}^{2n}\omega_{\left(i\right)}^{\left(m\right)}h\left[\chi_{k,(i)}^{}\right]\right\}{\left\{h\left[\chi_{k,(i)}^{}\right]\;-\;\sum_{i=0}^{2n}\omega_{\left(i\right)}^{\left(m\right)}h\left[\chi_{k,(i)}^{}\right]\right\}}^{T} \end{array}$$

where $r_{j}$ denotes the residual error, *N* is the window length for calculating the covariance of $r_{j}$, and $\chi_{j,(i)}^{}$ is the *i*-th *n*-dementional sigma point at step *j*. Besides, a 5-satellite selection based on GDOP are employed in ATC.

(3) Modified ATC (MATC)

Modified ATC (MATC) which uses the proposed satellite selection algorithm is also implemented to help to analyze the individual contributions of the satellite selection algorithm and RMNCE-UKF.

(4) CNR and satellite elevation based tightly-coupled integration (CNE-TC)

In a recently published article [25], satellites with elevations lower than 10° or CNR lower than 30 dB-Hz are excluded, and the variance on the pseudo-range estimates is weighted as:

(35) $${\hat{\sigma }}_{\rho }^{2}=\frac{a+b\cdot 10^{\frac{-\frac{C}{N_{0}}}{10}}}{\sin\left(Elev\right)}$$

where $\frac{C}{N_{0}}$ is the CNR value, Elev is the satellite elevation, *a* and *b* are empirical parameters, that are recommended to set *a* = 1 and *b* = 281^2^.

### 6.2. Semi-Physical Simulation Experiments

**Figure 4:**
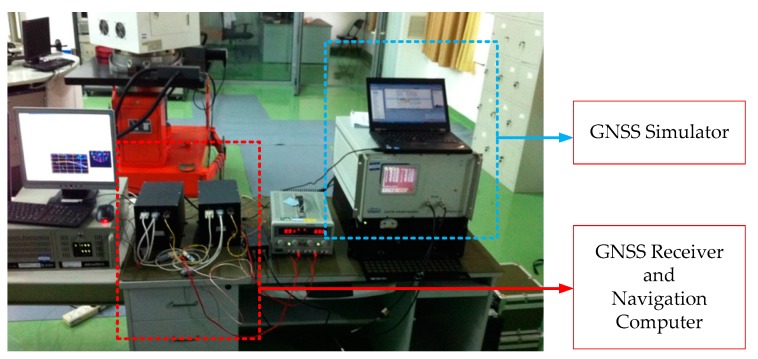
Devices employed in the semi-physical simulation experiments.

The CNR is difficult to be simulated without any personal bias, so STC, ATC and MATC are selected as the compared schemes to mainly verify the feasibility and effectiveness of the RMNCE-TC. The semi-physical experiment platform is shown in Figure 4. A trajectory generator is employed to produce the flight trajectory and corresponding true IMU data. Errors, such as bias, ARW, and VRW, are added to the true IMU data to simulate measured IMU data. Meanwhile, the barometer measurements are also simulated according to the true position data. All the sensor error settings can be found in Table 1. Furthermore, the Spirent GNSS simulator software suite SimGEN™ (Spirent Company, Sunnyvale, CA, USA) is employed to simulate GNSS data with 10 Hz output.

To evaluate the estimation accuracy of the RMNCE-TC algorithm, a time varying measurement noise deviation scheme was implemented. The standard deviation of the pseudo-range measurement noise was 1 m, except for: the period during the 730-th second to the 750-th second, where large errors were added to the satellites #10, #13, and #24 to simulate the multi-path effect; and the period during the 1900-th second to the 2500-th second, where all standard deviations were enlarged, particularly those of satellites #10, #13, and #24 were increased to 5m. The number of visible satellites and the detailed settings are listed in Table 2.

#### 6.2.1. Measurement Noise Variance Estimation

**Figure 5:**
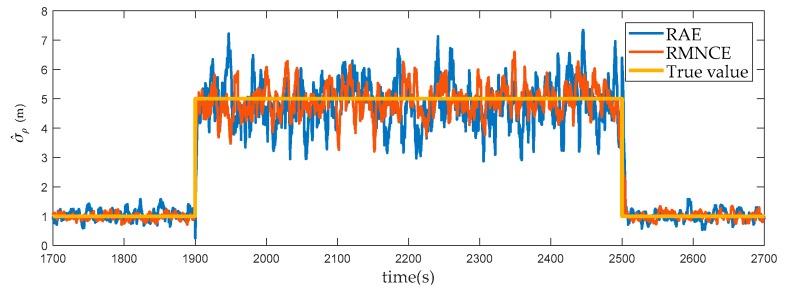
The estimated variances of the pseudo-range measurement noise obtained by RAE and RMNCE.

To verify the reliability of RMNCE, we compared the RMNCE results with those obtained by RAE. Figure 5 shows the results of satellite #24 which was always visible throughout the simulation. The comparison indicates that RMNCE is more robust and accurate than RAE, particularly when the true value of the measurement noise variance changes suddenly.

Besides, the error volatility of RMNCE is lower than RAE. We calculated the variances of the estimation errors during [1700 s, 1900 s], [1901 s, 2500 s] and [2501 s, 2700 s] intervals. The results of RMNCE are 0.0212, 0.2711, and 0.1651, and the corresponding results of RAE are 0.0304, 0.5828 and 0.3025. It is clear that RMNCE provides a more stable measurement noise variance estimation.

#### 6.2.2. Navigation Accuracy

Table 3 presents the root mean square errors (RMSE) of different navigation parameters of the entire simulation. The latitude and longitude errors are converted to northward and eastward position errors in meters. The RMCNE-TC owns an overall better performance than those of STC, ATC and MATC.

To further evaluate the navigation accuracy of the different frameworks, we compared the three-dimensional (3D) positioning error, which is defined as:

(36) $$d\left(k\right)\;=\;\sqrt{{\left[x_{E}\left(k\right)\;-\;x_{T}\left(k\right)\right]}^{2}\;+\;{\left[y_{E}\left(k\right)\;-\;y_{T}\left(k\right)\right]}^{2}\;+\;{\left[z_{E}\left(k\right)\;-\;z_{T}\left(k\right)\right]}^{2}}$$

where ${\left[x_{E}\left(k\right),y_{E}\left(k\right),z_{E}\left(k\right)\right]}^{T}$ is the ECEF position at the *k*-th epoch calculated by the different schemes, and ${\left[x_{T}\left(k\right),y_{T}\left(k\right),z_{T}\left(k\right)\right]}^{T}$ is the true ECEF position at the *k*-th epoch. In what follows, the 3D positioning errors obtained by the different schemes are analyzed over three typical segments, including [730 s, 750 s], [1900 s, 2500 s] and [2900 s, 2960 s].

(1) 3D Positioning Errors During [730 s, 750 s]

**Figure 6:**
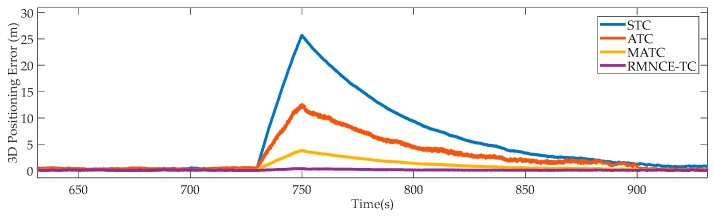
3D positioning errors of the selected schemes during [730 s, 750 s].

Figure 6 shows the positioning errors of the selected schemes from the 730-th second to the 750-th second, where the measurements of satellites #10, #13, and #24 were contaminated by large errors. The comparison result shows that RMNCE-TC is more robust in this scenario than other schemes. This superiority is mainly owing to the satellite selection procedure and the expanded **R** design. Table 4 lists the satellite selections, GDOP values, and positioning errors of these schemes at the 750-th epoch. In contrast to STC and ATC, the contaminated satellites #10, #13, and #24 are detected according to the threshold $\Delta \rho_{thrd}$ in MATC and RMNCE-TC. But the requirement for selecting five satellites necessitates including satellite #10 in the candidate list. Here, the expanding scale β employed in the adaptive UKF plays a vital role to effectively suppresses the negative impact of large error of satellite #10.

(2) 3D Positioning Errors During [1900 s, 2500 s]

**Figure 7:**
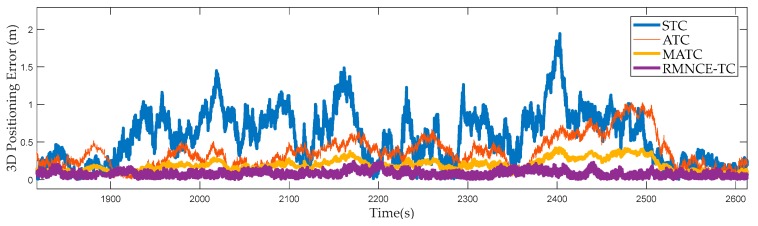
3D positioning errors of the selected schemes during [1900 s, 2500 s].

Figure 7 shows the positioning errors from the 1900-th second to the 2500-th second, during which the pseudo-range measurement noise was increased. Particularly the standard deviations of the pseudo-range measurement noise of satellites #10, #13, and #24, were set to 5 m. The fixed **R** employed in STC cannot adapt to the changes, which results in large positioning errors. ATC provides considerable improvement due to its adaptive strategy based on RAE. MATC provides a second smallest positioning error due to a better satellite selection. RMNCE-TC provides the smallest positioning error. Table 5 shows the GDOP of different schemes at the 2050-th second. Although the GDOP of RMNCE-TC is not the smallest, its 3D positioning error is the minimum.

(3) 3D Positioning Errors During [2900 s, 2960 s]

**Figure 8:**
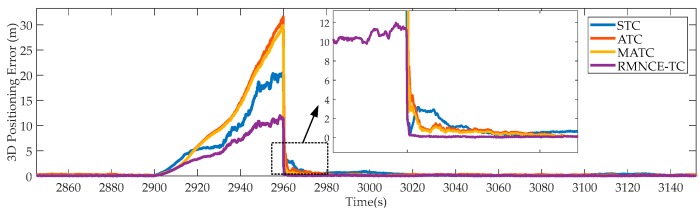
3D positioning errors of the selected schemes during [2900 s, 2960 s].

**Figure 9:**
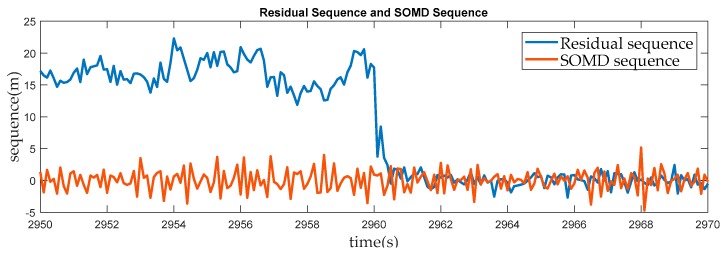
Comparison of the residual sequence and SOMD sequence of satellite #24 when the number of visible satellites changes from 1 to 8 at the 2960-th second.

Figure 8 presents the performances of the tested schemes when only one satellite is visible. The result shows that ATC and MATC even performs worse than STC, but RMNCE-TC still holds a better performance. Figure 9 compares the residual sequence and SOMD sequence of satellite #24. We note that the residual sequence is clearly biased during GNSS outage, which contradicts the conventional assumption that the residual sequence is zero mean white noise. Consequently, the RAE based **R** estimation becomes larger and generates harmful influence on the Kalman filter. In contrast, the SOMD sequence is more robust owing to its decoupling from the state estimation error. Hence, RMNCE-based noise estimation is more accurate than RAE.

### 6.3. Field Experiments

**Figure 10:**
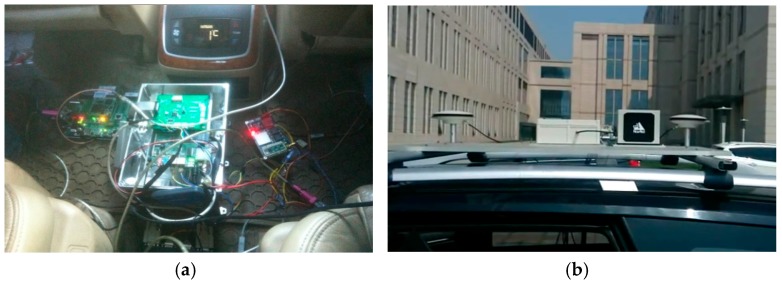
The test vehicle platform and equipment. (**a**) Designed hardware platform; (**b**) GNSS antennas and the NovAtel device.

A tightly-coupled integration system platform was designed and implemented within a vehicle to test the proposed architecture. The platform was mainly comprised of a Crossbow IMU-440 MEMS sensor (Milpitas, CA, USA), a differential GNSS receiver and a single chip MS5803 low-cost barometer, which is shown in Figure 10a. The performance indexes of the IMU-40 are listed in Table 6. Moreover, a NovAtel IMU-ISA-100C device (Calgary, AB, Canada) is utilized to provide high accuracy reference navigation solutions.

**Figure 11:**
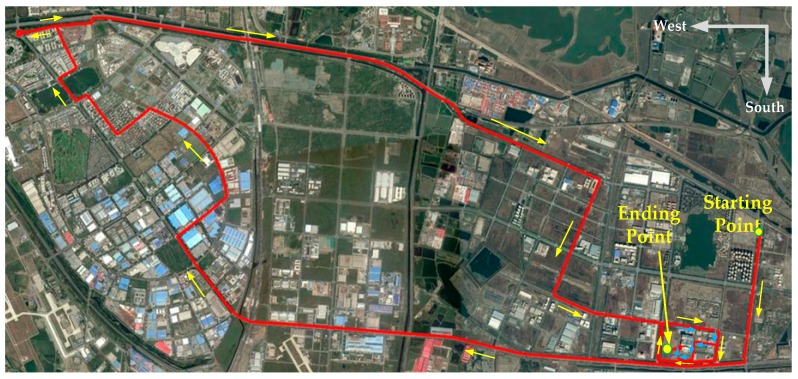
Reference trajectory of the field experiment (the blue arrows indicate the final part).

To make effective comparisons, GDOP based 4-satellite and 5-satellite selection standard tightly-coupled positioning schemes, denoted as STC4 and STC5 are carried out; and CNE-TC is also implemented to show the contributions of RMNCE-TC over the SNR/CNR and elevation based methods. More detail settings about the compared schemes are listed in Table 7. Different schemes during the test. The NovAtel reference trajectory is shown in Figure 11.

#### 6.3.1. General Evaluations

**Figure 12:**
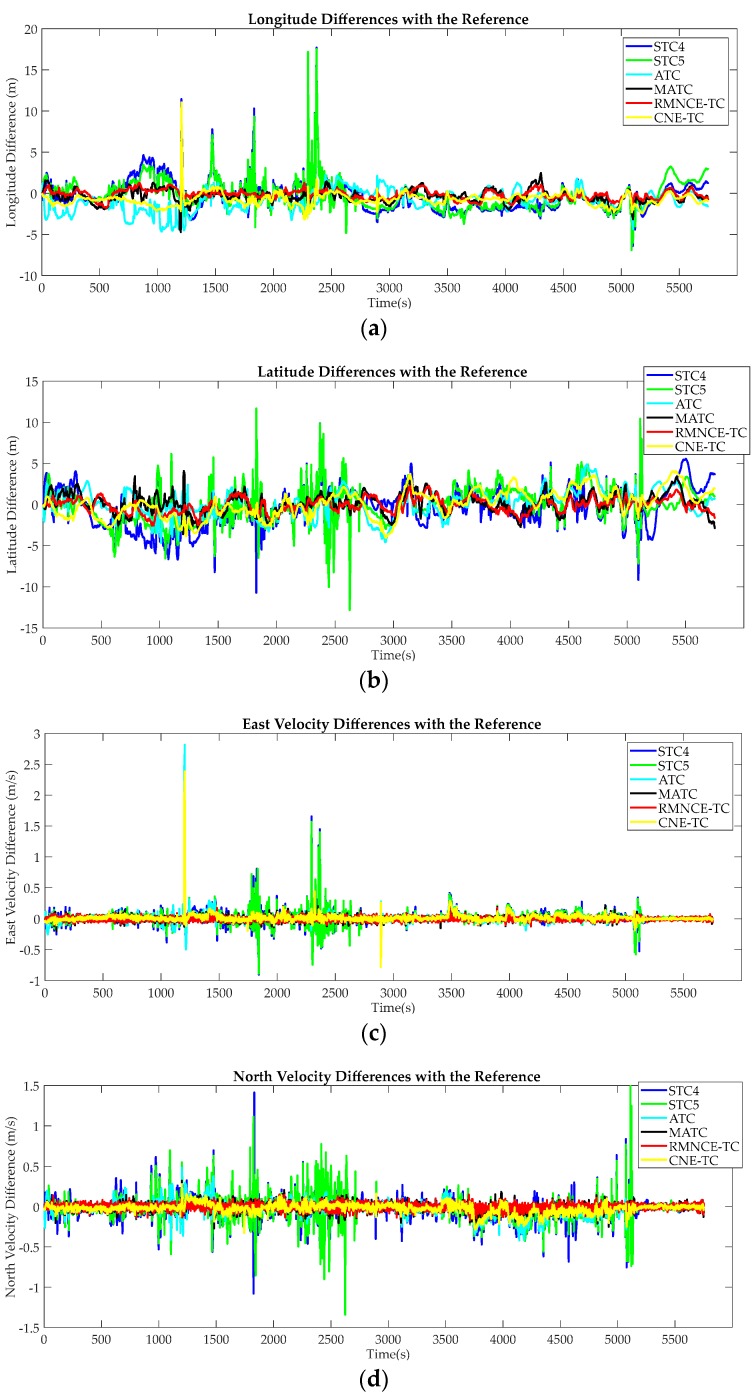
Navigation differences with respect to the reference trajectory using STC4, STC5, ATC, MATC RMNCE-TC and CNE-TC: (**a**) longitude differences (m); (**b**) latitude differences (m); (**c**) east velocity differences (m/s); (**d**) north velocity differences (m/s).

The main navigation errors of longitude, latitude, east and north velocities of all the considered schemes are shown in Figure 12. From Figure 12 we can find that: (1) in most cases, the accuracy of SCT5 is just marginally better than that of STC4 due to utilizing a redundant visible satellite. However, this may be counterproductive when the redundant measurements include large errors; (2) the performance of ATC is better than both STC4 and STC5 in most cases. However, when abnormal measurements occur, ATC suffers from large positioning errors; (3) MATC has an improvement over ATC owing to the RMNCE based satellite selection; (4) RMNCE-TC, which benefits from robust measurement noise estimation and the adaptive satellite selection, provides the best performance of all the considered schemes; (5) CNE-TC has an improvement over STCs and ATC, even performs better than RMNCE-TC at some epochs when the observability is good. But it still suffers from the large measurement errors when the observability quality becomes bad.

The RMSE was also employed to evaluate the global performances of the different schemes. Table 8 lists the RMSE results in detail. From the comparison, we note that MATC and CNE-TC have a similar navigation accuracy. RMNCE-TC provides the smallest navigation error.

#### 6.3.2. Navigation Reliability

**Figure 13:**
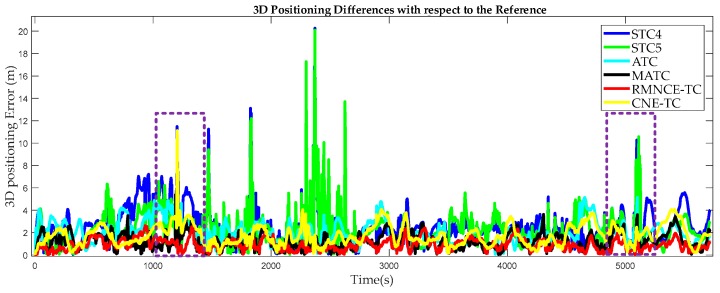
3D positioning differences of the test schemes with respect to the reference trajectory.

**Figure 14:**
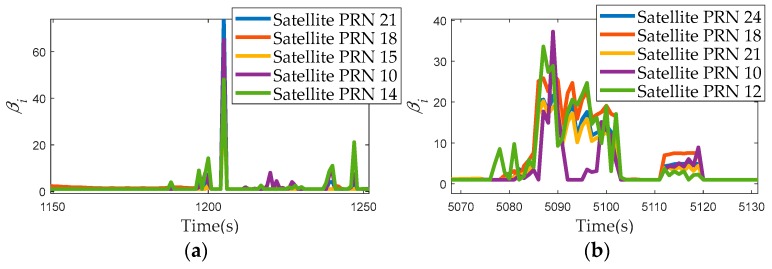
The value of the expanding scale $\beta_{i}$ with respect to the five pseudo-range measurements: (**a**) 1150 s to 1250 s; (**b**) 5070 s to 5120 s.

To evaluate the methods more effectively, 3D positioning error was calculated and statistically analyzed. The 3D positioning differences between the test schemes and the reference are shown in Figure 13. The positioning errors of STC4 and STC5 are much larger than those of others. CNE-TC performs better than STC4, STC5 and ATC. But when the environment becomes challenging, CNE-TC cannot cope with the large measurement errors effectively. MATC provides a smaller positioning error due to the RMNCE based satellite selection. RMNCE-TC performs best owing to not only the satellite selection algorithm but also the adaptive UKF. Figure 14 shows the expanding scales calculated by Equation (28) and we adopted the maximum as the final value at each epoch.

**Figure 15:**
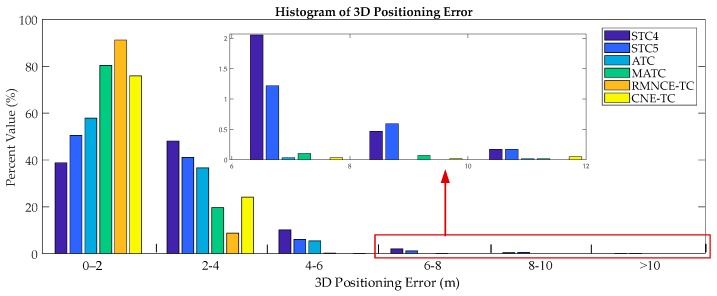
Histogram of the positioning errors obtained for the test schemes.

In practical applications, a position accuracy within 2 m is an important criterion for evaluating the reliability of a navigation solution [5]. Figure 15 shows the corresponding 3D error histograms. The percentages within 2 m are 38.79%, 50.48%, 57.35%, 80.36%, 91.23% and 76.88% for STC4, STC5, ATC, MATC, RMNCE-TC and CNE-TC respectively.

#### 6.3.3. Segment Analysis

**Figure 16:**
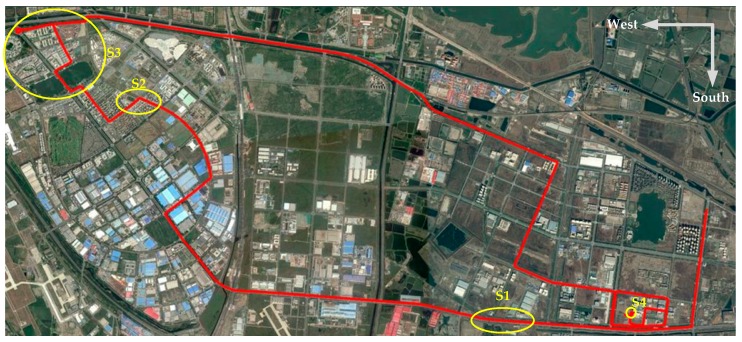
Four segments in the vehicle trajectory selected for detailed analyses.

**Figure 17:**
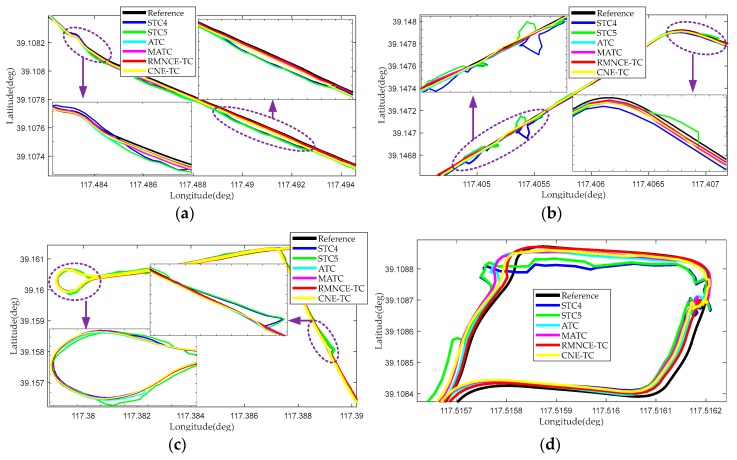
Local reference trajectories and those provided by different test schemes: (**a**) trajectories in segment S1; (**b**) trajectories in segment S2; (**c**) trajectories in segment S3; (**d**) trajectories in segment S4.

Four segments marked by yellow circles in Figure 16 were selected to elaborate on the comparison results. Figure 17 presents the trajectories provided by the different schemes. The trajectories obtained by RMNCE-TC reside the closest to the reference trajectories. But the performance of STC5 is not as good as might be expected when introducing a redundant satellite, and is at times even inferior to STC4 (e.g., see the results for S3 presented in Figure 17c). This indicates that merely increasing the number of satellites without introducing additional error handling methods can lead to unexpected degradation in the navigation performance. ATC provides an improved positioning accuracy due to the RAE-UKF. MATC and CNE-TC have an accuracy improvement over ATC but are still inferior to RMNCE-TC.

**Figure 18:**
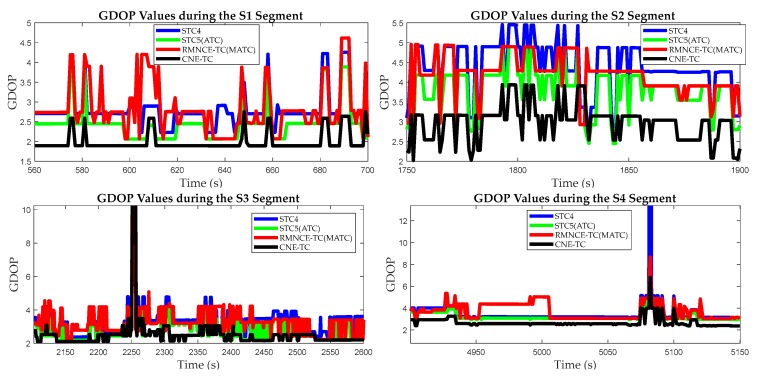
GDOP values of the different satellite selection algorithms during segments S1, S2, S3, and S4.

**Figure 19:**
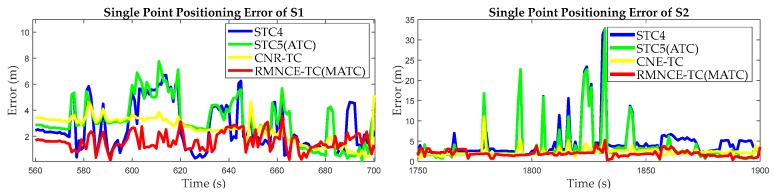

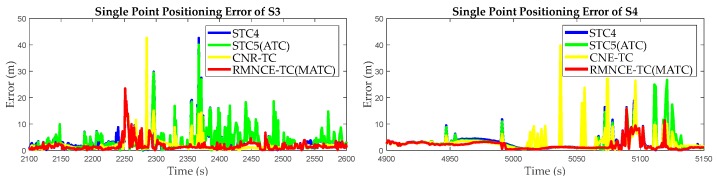
Single point positioning errors of the different satellite selection algorithms during segments S1, S2, S3, and S4.

The GDOP values obtained by STC4, STC5 (with an equivalent satellite selection as that of ATC), RMNCE-TC (with an equivalent satellite selection as that of MATC) and CNE-TC are plotted in Figure 18. The results show: the GDOP values of STC5(ATC) calculated using five satellites are lower than STC4 and RMNCE-TC (MATC); CNE-TC always provides a minimum GDOP value due to its loosely excluding condition; the GDOP of RMNCE-TC is relatively large at many epochs. We calculated the single point positioning (SPP) errors of the selected satellite measurements, which are shown in Figure 19. Unexpectedly, the SPP error of STC5(ATC) is very large, and RMNCE-based satellite selection presents the best performance. This further indicates that adding redundant satellites always decreases the GDOP value, but may not always improve the positioning accuracy. Increasing the quantity of observations has the risk of unexpectedly introducing large measurement errors into the system. Therefore, the measurement quality should be fully considered when selecting an appropriate satellite combination.

**Figure 20:**
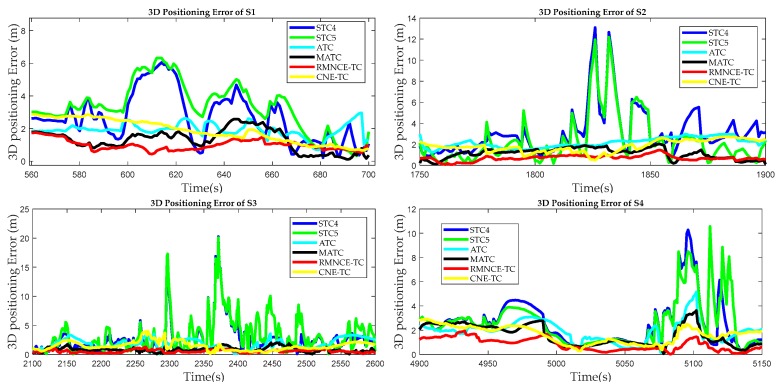
3D positioning errors of the different test schemes during segments S1, S2, S3, and S4.

Finally, the 3D positioning errors during segments S1, S2, S3, and S4 are presented in Figure 20. CNE-TC has the ability to control the large errors based on the weighted pseudo-range variance, but it is still inferior to RMNCE-TC. RMNCE-TC can adaptively eliminate the satellites with poor measurement quality, and effectively estimate **R** to obtain a robust solution.

## 7. Conclusions

In this paper, we introduce a novel adaptive low-cost INS/GNSS tightly-coupled integration architecture that can provide reliable navigation solutions within disturbed GNSS communication environments. The proposed architecture features an adaptive redundant measurement noise covariance estimation (RMNCE) approach, which is characterized by only employing the measurement system information. Different from traditional algorithms, this method avoids the effect of Kalman filter state vector estimation error. The RMNCE approach is applied to design a fast satellite selection algorithm and an adaptive UKF in our proposed system, which has increasingly improved system performance and accuracy. Both semi-physical simulation and field experiments have been carried out to demonstrate its overall better performance compared to the standard tightly-coupled schemes, RAE-based adaptive scheme, and CNR and satellite elevation-based adaptive tightly-coupled integration scheme. The experimental results lead us to conclude that: (1) the RMNCE approach can achieve a comprehensive better and robust measurement noise estimation results than the traditional noise estimation algorithms; (2) the RMNCE-based satellite selection algorithm takes both measurement noise and GDOP into consideration to derive an optimal satellites combination, and hence can avoid the risk of unexpectedly introducing large measurement errors into the system; (3) the RMNCE-based adaptive UKF is advantageous in reducing the positioning errors when the GNSS measurements are contaminated; (4) the proposed architecture effectively limits positioning errors when the GNSS measurement quality is poor, and can provide 91.2% positioning reliability (2 m positioning error), which performs the best among all of the test schemes.

## Figures and Tables

**Table 1:** Sensor error settings employed in the simulation experiments.

Parameters	Performance
Gyroscope bias	10 °/h
Angle random walk	$0.3\;{}^{\circ}/\sqrt{h}$
Accelerometer bias	1 mg
Velocity random walk	$1\;\mathrm{mg}/\sqrt{\mathrm{Hz}}$
Variance of barometer	25 m^2^

**Table 2:** The GNSS measurement error settings.

Time(s)	Satellite Number	$\sigma_{\rho }\left(m\right)$	$\sigma_{\dot{\rho }}\left(m/s\right)$	Special Settings
730–750	7	1	0.01	add additional large errors ^1^ to the #10, #13 and #24 satellite pesudo-range easurements
1900–2500	7	2	0.01	increase $\sigma_{\rho }$ of #10, #13 and # 24 to 5m
2900–2960	1	1	0.01	only #24 is visible
other	8	1	0.01	—

^1^ The additional large errors of the pseudo-range measurements of satellites #10, #13, and #24 are set as time varying, which can be expressed as error = *a*∙*t* + *b*, where *a* = 1, *b* = 100, 90, and 80, respectively.

**Table 3:** RMSE obtained by the different schemes.

	STC	ATC	MATC	RMNCE-TC
Latitude (m)	3.0236	0.6926	0.5960	0.3706
Longitude (m)	3.8596	1.5556	1.4370	1.1603
East velocity (m/s)	0.1528	0.0797	0.0767	0.0698
North velocity (m/s)	0.1795	0.0863	0.0864	0.0866
Heading (°)	0.7027	0.4813	0.4766	0.4656
Pitch (°)	0.4743	0.2013	0.1714	0.1015
Roll (°)	0.1070	0.1214	0.1176	0.1088

**Table 4:** Satellite selection results of the different schemes at the 740-th second.

	Selected Satellites ID	GDOP	3D Positioning Error
STC	10, 13, 29, 21	2.766	13.580 m
ATC	15, 10, 18, 13, 29	2.408	3.115 m
MATC	15, 10, 18, 21, 29	2.452	2.215 m
RMNCE-TC	15, 10, 18, 21, 29	2.452	0.152 m

**Table 5:** Satellite selection results of the different schemes at the 2050-th second.

	Selected Satellites ID	GDOP	3D Positioning Error
STC	10, 13, 21, 29	2.430	1.072 m
ATC	10, 13, 18, 24, 29	2.159	0.728 m
MATC	15, 10, 18, 21, 29	2.168	0.461 m
RMNCE-TC	15, 10, 18, 21, 29	2.168	0.205 m

**Table 6:** The performance parameters of the IMU sensor.

Gyroscope Performance	Accelerometer Performance
Bias stability: 10 °/h	Bias stability: 1 mg
ARW: $4.5\;{}^{\circ}/\sqrt{h}$	VRW: $0.5\;m/s/\sqrt{h}$
Input range: ± 200°/s	Input range: ±10 g
Scale factor non-linearity: ≤100 ppm	Scale factor non-linearity: ≤100 ppm

**Table 7:** Different schemes during the test.

Label	Satellite Selection	Filer Technique
STC4	4 satellites, DGOP based	Standard UKF
STC5	5 satellites, DGOP based	Standard UKF
ATC	5 satellites, DGOP based	RAE-UKF
MATC	5 satellites, RMNCE based	RAE-UKF
RMNCE-TC	5 satellites, RMNCE based	RMNCE-UKF
CNE-TC	Variable selected satellite number based on CNR and elevation	R is weighted by CNR and satellite elevation

**Table 8:** Global RMSE values of the different schemes.

	STC4	STC5	ATC	MATC	RMNCE-TC	CNE-TC
Latitude (m)	1.8205	1.7114	1.3430	1.1557	0.5697	1.5629
Longitude (m)	2.2633	2.1034	1.7706	1.4231	0.8689	1.1514
East velocity (m/s)	0.1124	0.1078	0.0595	0.0301	0.0299	0.0300
North velocity (m/s)	0.1122	0.1337	0.0406	0.0382	0.0370	0.0475
Heading (°)	0.9092	0.8389	0.6072	0.6101	0.6061	0.6001
Pitch (°)	0.2838	0.2537	0.1925	0.1905	0.1835	0.1921
Roll (°)	0.1957	0.1832	0.1852	0.1860	0.1852	0.1858
